# Root System Water Consumption Pattern Identification on Time Series Data

**DOI:** 10.3390/s17061410

**Published:** 2017-06-16

**Authors:** Manuel Figueroa, Christopher Pope

**Affiliations:** Telefonica Investigación y Desarrollo Chile, Manuel Montt 1404, 7501105 Santiago, Chile; manuel.figueroa@alumnos.usm.cl

**Keywords:** time series analysis, precision agriculture, data science, pattern recognition, Internet of Things

## Abstract

In agriculture, soil and meteorological sensors are used along low power networks to capture data, which allows for optimal resource usage and minimizing environmental impact. This study uses time series analysis methods for outliers’ detection and pattern recognition on soil moisture sensor data to identify irrigation and consumption patterns and to improve a soil moisture prediction and irrigation system. This study compares three new algorithms with the current detection technique in the project; the results greatly decrease the number of false positives detected. The best result is obtained by the Series Strings Comparison (SSC) algorithm averaging a precision of 0.872 on the testing sets, vastly improving the current system’s 0.348 precision.

## 1. Introduction

The main objective of the study is to automatically identify the Root System Water Consumption (RSWC) pattern in soil moisture time series data. This study is part of an irrigation efficiency project that uses soil moisture sensor data to generate a moisture prediction to recommend an irrigation schedule.

Water usage in agriculture accounts for approximately 87% of water usage in many countries [1]. Therefore, understanding how water works and to achieve an efficient usage of it are fundamental tasks. In agriculture, irrigation accounts for around 70% of crops’ development [2,3]. The correct usage of water may yield bigger production and better fruit quality. Therefore, understanding how water works is a fundamental task. Using soil sensors [4], it is possible to observe how water behaves around plant roots. To maximize returns and minimize environmental impact on agriculture, data from soil and meteorological sensors are analyzed to know when to water crops and how much water should be used; these analysis techniques are normally referred to as precision agriculture [2,3].

Soil moisture sensors allow agronomists to understand how water is being consumed by crops; they can also display the way water interacts with soil and crop roots. This understanding, combined with observable on-field plant reactions, helps agronomists decide when and how to irrigate. Most agronomists base their irrigation decisions on the traditional evapotranspiration formula [5,6,7,8,9], based mainly on climatic factors.

Using soil sensors, it is possible to observe a plant’s reaction to irrigation and learn about soil drainage. This creates an expected pattern generated by soil moisture during and after irrigation, and it is referred to as the Root System Water Consumption (RSWC) pattern. A non-conforming section of the time series to the RSWC pattern may alert agronomists of the possible malfunction of sensors or a soil moisture/crop interaction change.

The detection of RSWC is a problem where manual identification of these patterns in the data is possible, but very time consuming [2,3]. Unsupervised learning techniques are created to work without a training set, and manually-labeled ground truth sets are used to build supervised learning algorithms and to assess each algorithm’s classification performance.

The state of the art is summarized in a survey by Gupta et al. [10], explaining many of the challenges and how to face them when looking for outlier subseries in time series data. The very definition of an outlier sub-series is a problem in itself, picking a similarity function that’s appropriate for the data or the length a subseries should have are some examples of challenges that may be faced. The problem addressed in this study is closely related to outlier patterns’ detection, as non-RSWC-compliant sections within the time series data may be considered outlier subseries.

For studying outlier subsequences in time series, early work by Keogh et al. [11] utilizes Markov models and suffix trees to find patterns in a time series and to predict the likelihood of a new pattern. Bu et al. [12] work to find the top-K most unusual subseries within a time series applying the Haar Wavelet transform. Outstanding work on time series is presented by Lin et al. [13]; they created a method to reduce dimensionality and discretize data into a short string that allows quick and efficient sub-series comparisons, creating a lower bound for Euclidean distance. The work is further expanded by Keogh et al. [14] introducing the concept of series discord as the most unusual subsequence on a time series and a HOT SAX algorithm that uses the strings to index the data and efficiently find the outlying sequence.

The HOT SAX algorithm has various uses; one of the most common is to detect anomalies on ECG scans [15,16], but it has also been expanded to work on shape detection using a one-dimensional representation to find the most unusual shape within a dataset [17]. Senin et al. [18] propose using the sequitur algorithm [19] to compress the discretized time series using context-free grammars [20] and creating a set of rules. These grammar rules provide a hierarchical structure that can be mapped back to the original time series points, and utilizing the frequency of the rules’ occurrence on the series, discord subsequences can be identified accurately and more efficiently than in previous work.

The techniques provided by these authors are used to propose three novel algorithms: Top Rule Pattern (TRP), pre-Validated Top Rule Pattern (preTRP) and Series String Comparison (SSC); the specifics of the methods and developed algorithms will be presented in the Materials and Methods section.

Data from five fields, covering three different species of crops and spanning from early 2014 until late 2016, are considered for this study. These data are obtained from Sentek EnviroSCAN FDR (Frequency Domain Reflectometry) probes [4] placed at each field; soil moisture is measured every 15 min at five depths (20, 40, 60, 80 and 100 cm), the aggregation of these measurements generates a data point on the time series.

The rest of the paper is organized as follows. Section 2 explains the RSWC pattern; Section 3 explains the basic concepts of time series analysis, shows a summary of the datasets used and proposes algorithms to solve the problem at hand. In Section 4, the results of the different algorithms are exposed to be discussed and compared with the current DHC algorithm. Finally, Section 5 offers some discussion on the study results.

## 2. Root System Water Consumption Pattern

The time series data generated out of soil moisture measurements are expected to produce a pattern for each irrigation event. This pattern helps agronomists observe the behavior of moisture through soil and the interaction between soil, water and crop water consumption. This pattern will be referred to as the Root System Water Consumption pattern (RSWC).

**Figure 1:**
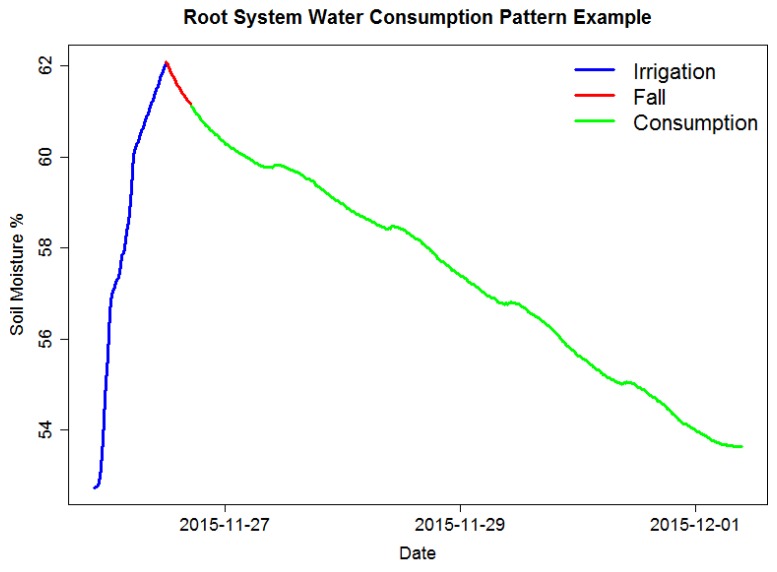
RSWC pattern example. The pattern consists of three segments: irrigation, fall and consumption.

To identify RSWC, soil moisture time series data must be divided into sections. Sections refer to irrigation events that could have happened whether by human intervention like irrigation or by rainfall. Figure 1 shows an example of the RSWC pattern and its corresponding segments. Sections are composed of three distinctive segments:

- Irrigation refers to the time series segment where soil moisture increases through time. This is mainly because soil is being irrigated; in some cases, it can also be due to rain or excessive air humidity. This segment usually lasts around 6 to 12 h depending on the field irrigation schedule.
- Fall refers to the time series segment after an irrigation event, where soil moisture decreases rapidly at a pace higher than during consumption. This segment may not be present in every section, since it depends on whether soil humidity has surpassed the saturation level or not. This segment usually lasts a couple of hours.
- Consumption refers to the time series segment after an irrigation or fall where soil moisture decreases in a much slower rate than the fall segment. This decrement is mainly due to crops’ water consumption and also due to evapotranspiration. This segment usually lasts several days. It is possible to identify this segment visually because it usually shows a ladder step shape.

**Figure 2:**
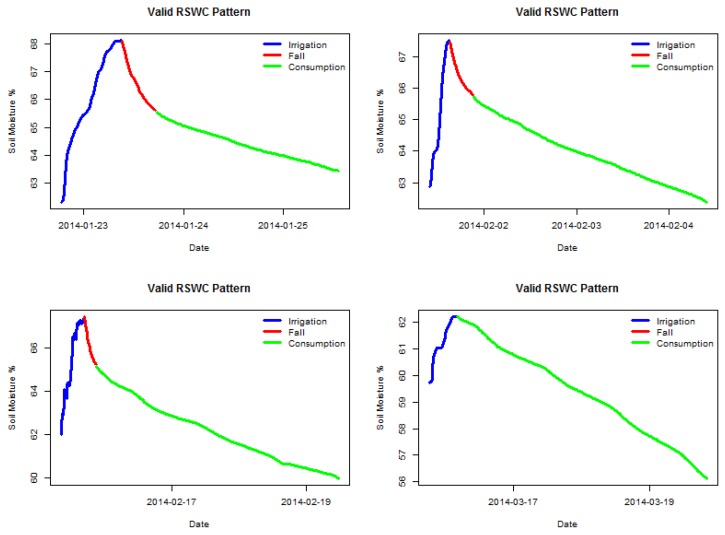
Examples sections with valid RSWC pattern.

**Figure 3:**
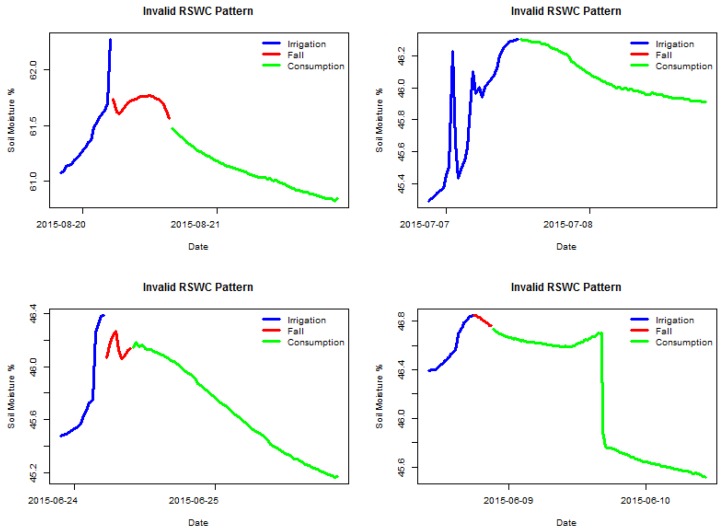
Examples sections with invalid RSWC pattern.

Figure 2 shows four examples of valid labeled sections, and Figure 3 shows four examples of invalid labeled sections. It is possible to observe which sensor behavior is considered valid, a steady soil moisture increase for the irrigation segment followed by either a fall or a consumption segment. The two latter segments must show a decreasing trend. All segments must be clearly connected, meaning no anomalous gaps between measures. The following sections have been manually selected by an expert visualizing the time series data.

**Figure 4:**
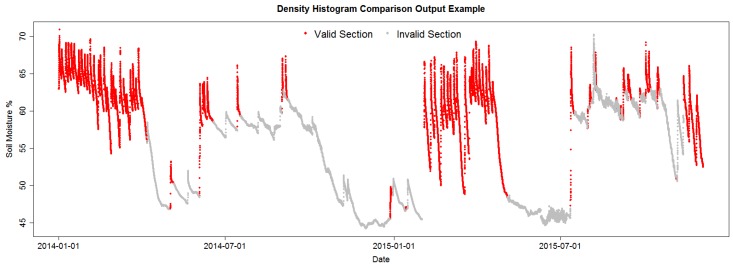
DHC algorithm output example.

Figure 4 shows the pattern identified in the data by the current identification algorithm (DHC) in the project; some patterns are clearly misidentified as valid, but the image shows the RSWC pattern in a proper context, valid RSWC sections are colored in red and invalid sections in gray. The correct identification of the RSWC patterns aids agronomists to quickly visualize situations where sensor measurements are within the expected behavior (valid) and when not (invalid). Invalid sections may happen when sensors malfunction or when irrigation patterns change abruptly, for example a water irrigation line was broken by field machinery resulting in the field getting over irrigated; or the field is starting to dry up, low soil moisture. These scenarios may trigger alarms to agronomists in order to fix any anomalous situation. Furthermore, a correct identification of valid and invalid RSWC will generate a useful database for future sensor behavior prediction. This is the objective of our irrigation efficiency project: prediction of soil moisture for irrigation scheduling.

## 3. Materials and Methods

### 3.1. Datasets

Data are obtained from five Sentek EnviroSCAN FDR probes [4] placed on fields of avocados, kiwis and nectarines. Measurements are captured every 15 min at 5 different depths: 20, 40, 60, 80 and 100 cm. Each field has one sensor probe that may represent several acres. Even though each sensor probe is only able to capture soil moisture in its nearby area, a few centimeters, it is considered to represent the overall moisture of a field. Each measurement corresponds to the available water content at a particular depth. These measurements range from 0 to 100, were 0 means there is no available water and 100 means soil is saturated with water that is usable for the crop. Sensors are calibrated on installation to represent this 0 to 100 range depending on factors such as soil structure, salinity and crop roots’ mechanics.

Measurements are aggregated using the sum of all depths and rescaling them to a 0 to 100 range, equivalent to calculating the mean of all depths. This aggregation is considered to better represent total available water at multiple depths [2,3].

**Figure 5:**
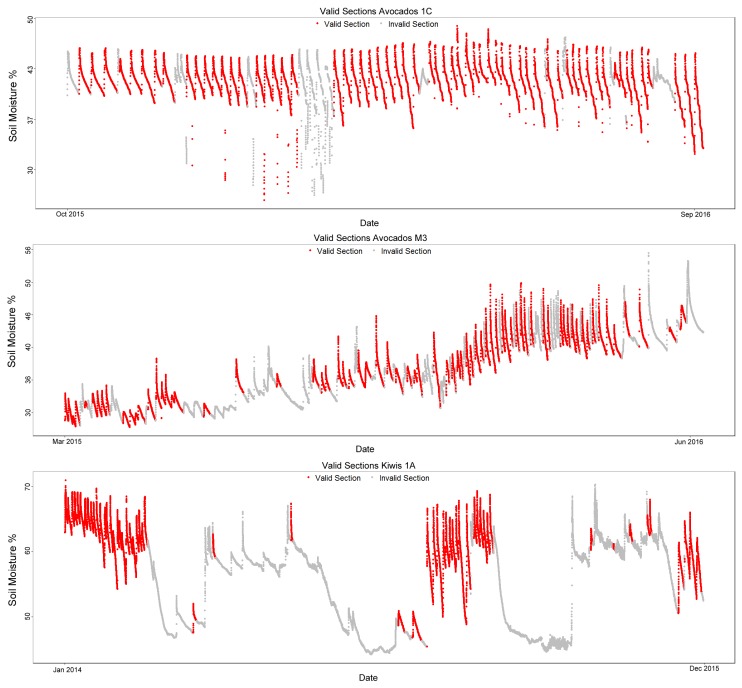
Plots of the training datasets: Avocados 1C, Avocados M3 and Kiwis 1A.

The datasets are split into training and testing datasets, 3 for training and 2 for testing. Training datasets were used to tune the algorithm parameters, and the testing datasets were used for validation. For each dataset section, an expert manually labeled the section as valid or invalid with the RSWC pattern, thus generating a ground truth. This ground truth will be used to assess each method’s performance. Figure 5 shows the data with the manually-labeled valid sections colored in red and invalid sections in gray. Table 1 is a summary of the datasets.

The datasets span from 291 days on the smallest dataset to a little under 2 years on the Kiwis 1A data. Table 1 columns show the number of total, valid and invalid sections on each dataset that should be classified. Finally, the last column, “pre-classified”, is the number of sections that can be discarded as invalid based on three basic rules of the RSWC pattern that are discussed in the following sections.

On all datasets, most of the invalid sections can be pre-classified using the basic rules; this is mostly due to very short patterns (under a 2-day duration) being detected as sections by the slope analysis algorithm. Despite these short sections, manual verification confirms that the RSWC patterns are present.

**Figure 6:**
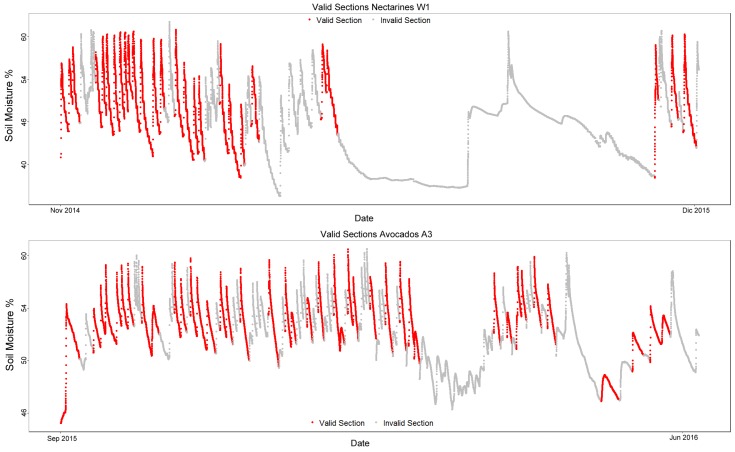
Plots of the testing datasets: Nectarines W1 and Avocados A3.

There are two testing sets that were used to validate the results obtained, Nectarines W1 and Avocados A3. The nectarine field tests the algorithm on a different kind of crop, and another avocado field allows for confirmation on a dataset similar to the training ones. Figure 6 and Table 2 show the testing datasets.

### 3.2. Time Series Section Identification

**Figure 7:**
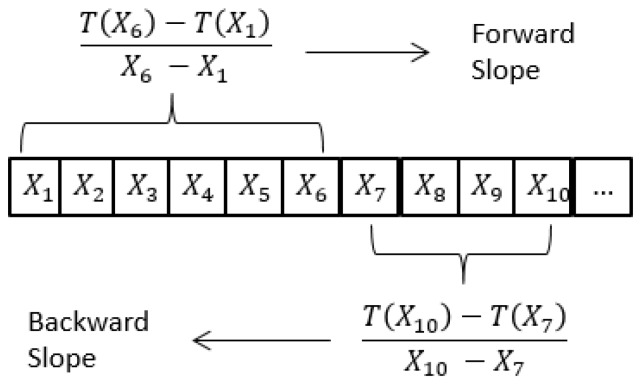
Forward and backward slope calculation.

The first step for all algorithms created is to partition the time series into sections. To achieve this, slopes are calculated in the following fashion; forward every 5 data points and backwards every 3, as shown in Figure 7. These slopes are calculated for the whole time series. Then, data points must be labeled as one of the three segments previously described. Data points with a positive forward slope and a negative backward one are labeled as irrigation. All remaining points are labeled temporarily as consumption. Within a series of consecutive consumption data points, their forward slope is analyzed to identify outliers. Data points with a highly negative forward slope or highly positive backward slope are labeled as fall.

**Figure 8:**
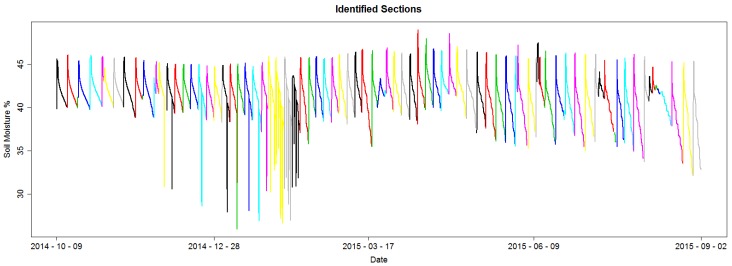
Example of identified sections; each section has a distinct color to differentiate from its adjacent sections.

A section is identified as all data points within consecutive valleys and containing an irrigation, fall and consumption segment. Finally, each section is given a unique identification number. Figure 8 shows the identified sections for an example dataset; each section is colored to differentiate from its adjacent sections.

**Figure 9:**
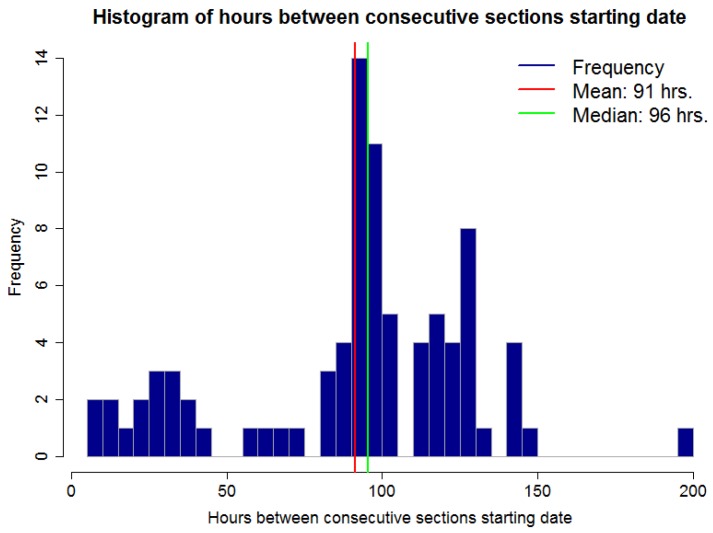
Hours between consecutive sections starting point.

Figure 9 shows a histogram for the number of hours between two consecutive section starting points for the dataset presented in Figure 8. In other words, the duration for a section. It is possible to observe that this time is not regular. Some sections span a few hours, under 48 h or 2 days, and others span for over 100 h or over 4 days. This exemplifies one of the difficulties of the RSWC pattern; the duration of the pattern is not constant. An irregular duration may be due to irrigation schedule changes because of water restrictions, such as water or labor availability, because of rain fall or sensor malfunction.

### 3.3. Current Algorithm: Density Histogram Comparison

To validate sections, a novel density histogram comparison (DHC) algorithm has been implemented. This algorithm is implemented currently at our water efficiency project. First, all sections are filtered by a set of three basic rules; this generates a pre-classification. A section must comply with all three rules in order to be considered as a candidate of a valid RSWC pattern:

- Section duration is between 2 and 10 days.
- The soil moisture range (max-min of the section) is greater than 1.
- The irrigation segment is over 2 h.

**Figure 10:**
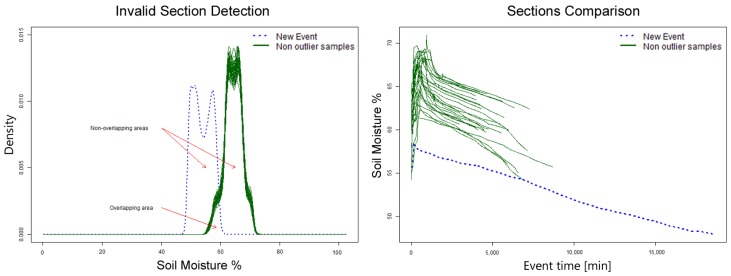
Example of DHC algorithm results.

First, a density histogram based on soil moisture is calculated for each section and compared with a sample of 50 valid patterns. Each section’s histogram has an area under the curve of 1; the non-overlapping area is calculated between the section being verified and the sample. The value for this area is of 2 if they do not overlap and 0 if they are identical. If the average non-overlapping area of the 50 samples is 1.5 or higher, then the section is considered invalid. Sections with an area under 1.5 are valid and added to the set of valid patterns. An example of this algorithm execution is shown in Figure 10; the new section (blue dotted line) will be labeled as invalid.

The DHC algorithm requires some sections previously defined as valid in order to have a reference set to which to compare new sections, as shown in Figure 10. This algorithm focuses mainly on classifying sections as valid whenever their soil moisture range is within the already valid sections’ range. This is an important disadvantage for this method, as it relies on a constant soil moisture range. This is usually not the case for most crops as agronomist may schedule irrigation at lower soil moisture levels, inducing water stress to crops or increase soil moisture level in case crops require more water depending on their phenological stage [2,3,6].

### 3.4. New Validation Algorithms

Three novel algorithms were developed to identify and validate the RSWC pattern. This section will introduce the methods used by the algorithms to analyze the time series data, and it follows with the description of each proposed algorithm.

#### 3.4.1. Time Series Analysis Methods

The methods presented here are necessary to understand the algorithms developed, and they explain some basic time series analysis methods and notations. A time series consists of an ordered set of real valued variables; in this case each value represents the soil moisture levels at the corresponding time. The notation used comes from Keogh et al. [14]; a time series $T=t_{1},\cdots ,t_{m}$ is an ordered set of *m* variables.

To compare sequences easily, two methods are used to reduce dimensionality and to obtain a discrete representation of the sequence. The first method is Piecewise Aggregation Approximation (PAA) [21]; it is used to represent an *m*-dimensional series in *w* dimensions; the *m* values are grouped into *w* vectors; and the mean of each of them is used as a value in the new representation. $\bar{X}={\bar{x}}_{1},{\bar{x}}_{2},\ldots ,{\bar{x}}_{w}$ where each ${\bar{x}}_{i}$ is calculated as shown in (1) is the PAA representation of an *m*-dimensional series *T*.

(1) $${\bar{x}}_{i}=\frac{w}{m}\sum_{j=\frac{m}{w}\left(i-1\right)+1}^{\frac{m}{w}i}x_{j}.$$

**Figure 11:**
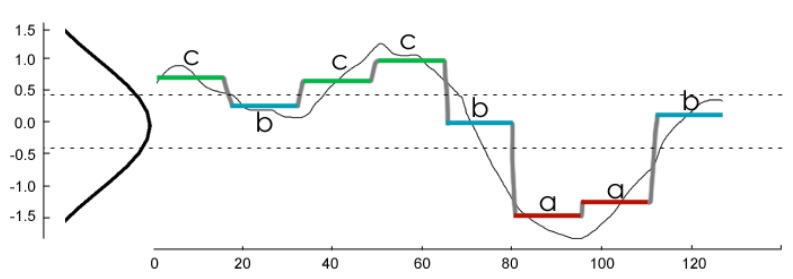
PAA/SAX representation.

A discrete representation is obtained from the PAA form utilizing a Symbolic Aggregate ApproXimation (SAX) [22]; the method transforms the PAA series into a word with *w* symbols where each symbol comes from an α-sized alphabet. In Figure 11, the original time series of 128 values is represented by a thin black line; the series is reduced to 8 PAA vector values, and each is assigned a letter from the alphabet {a,b,c} resulting in the word “cbccbaab”.

The symbols are assigned with equiprobability; if the original series is standardized by subtracting the mean value and dividing by the standard deviation, a N(0,1) distribution can be used to obtain breakpoints for the probability of each letter. Table 3 shows the breakpoints for alphabet sizes α from 3 to 5; in the figure example, the values lower than −0.43 are assigned the symbol “a”; between −0.43 and 0.43 are assigned “b”; and values over 0.43 get “c”.

SAX representation allows quick comparison of time series sub-sequences; the method used to determine whether two subseries correspond to the same pattern is mindist comparison [18]; two SAX words are considered mindist equivalent if each symbol has 1 or less distance from the corresponding symbol on the other word. Using mindist, the words “aabb“ and “aacc” are equivalent, but “aabb” and “aabd” are not.

An entire time series *T* can be discretized by extracting all possible sub-sequences of length *p* using a sliding time window; starting from the first position of *T*, a subsequence $C_{1}=t_{1},\cdots ,t_{p}$ of length *p* is extracted, then the window moves to the next position extracting the sequence $C_{2}=t_{2},\cdots ,t_{p}+1$, and so on, until the last position matches $t_{m}$.

Finally, the last method necessary for the algorithms comes from Senin et al. [18]; it uses the set of words extracted from a time series to create a corresponding context free grammar; this organizes the words using a hierarchical structure that uses grammar rules to help identify which patterns of words are most common and which words do not follow any patterns in the time series.

The algorithm RePair [23] is used to extract the grammar rules from the SAX representation of the time series; the algorithm identifies repeating pairs of symbols and assigns rules to compress a string of characters. Each SAX word extracted using the time window method is considered a symbol for the grammar, and the rules generated by RePair are directly related to the frequency of patterns on the original time series.

Combining these methods, three algorithms are proposed to identify the RSWC pattern on the time series data. The first two algorithms, Top Rule Patterns (TRP) and pre-validated Top Rule Patterns (preTRP), are unsupervised algorithms that use grammar rules’ coverage to identify the most common patterns. The third algorithm is called Series String Comparison (SSC); it uses a very simple method to compare SAX words to a reference set of valid patterns to classify new series. SSC changes the paradigm of the solution, using a supervised algorithm on an originally unsupervised problem. It uses RSWC patterns from different fields to support its decision making.

#### 3.4.2. Top Rule Patterns and Pre-Validated Top Rule Patterns

TRP takes a time series and three parameters to classify the sections identified with the slope analysis method from Section 3.2. The first parameter used is *w*, which represents the window size for the sliding window; this translates to how many points in the series will be covered by each SAX word. The other two parameters are *a* and *p*; the first one is the size of the alphabet for the SAX words, and *p* is the PAA vector size, corresponding to the length of each word.

TRP also uses a binary variable preTRP to differentiate two variants of the algorithm. preTRP checks for the three basic rules, explained in Section 3.3, on each time series section before processing them, while TRP performs this verification at the end. Pseudo-code for the TRP algorithm is shown in Algorithm 1. The process starts by using the slope analysis to partition the time series into sections.

**Algorithm 1** Top rule patterns.1:**function** (TRP〈T,w,a,p,preTRP〉):
2:  valid_sections = list()
3:  sections = slope_analysis(T)▹ Returns the partitioned series4:  **if** preTRP **then**
5:   T = concatenate(basic_rules(sections))
6:   sections = standardize(sections)
7:  **end if**
8:  grammar = SAX_grammar(T,w,a,p)
9:  **for** section in sections **do**
10:   **if** basic_rules(section) **then**
11:    mean_coverage = get_coverage(section, grammar)
12:    **if** mean_coverage ≥ 3.0 **then**
13:     valid_sections.append(section.id)
14:    **end if**
15:   **end if**
16:  **end for**
17:  **return** (valid_sections)
18:**end function**


Then, there is a split for the two variants of the algorithm. If the variable *preTRP* is one, the three basic rules are checked, and all of the sections that do not fulfill them are discarded and labeled as invalid. The sections that do follow the basic rules are standardized using the section’s mean and standard deviation to reduce linear discontinuities when they are concatenated together; this process is important because these discontinuities have great effect on the patterns if left untouched. For TRP, when the variable *preTRP* is zero, the basic rules for the sections are checked at the end of the process.

With the series partitioned into sections and reduced to only pre-validated sections in the case of preTRP, the series is discretized into SAX words with the sliding time window method, and a grammar is generated with RePair. The grammar is a set of rules and can be mapped directly to each point in the data; if a rule covers a SAX word, then all of the points the word was made with are covered by the rule.

For each section, a score is calculated based on the number of rules that cover it and the importance of said rules. The rules are organized into quantiles so that the rules that cover the most points are part of the 4th quantile, and the ones that cover the least amount of data are in the 1st one. Each point in the time series is assigned a number from 0 to 4; 0 means that no rules cover it, and 1 to 4 are assigned according to the quantile of the best rule that covers the point. The score for a section is the average of the scores of its points.

Finally, sections are classified using the “mean_coverage” score; if it is 3 or higher and they fulfill the basic rules, then the section is considered a valid RSWC pattern. The score is directly related to the rules that cover the section; a score of 3 means that most points in the section are considered in the 3rd or 4th quantile rules; thus, they are among the most common patterns in the series.

#### 3.4.3. Series String Comparison

The third proposed algorithm, SSC, is similar to the DHC algorithm, but it uses a different method to compare sections. The initiation is similar to preTRP; the time series is partitioned into sections, and the ones that do not follow the basic rules are immediately labeled as invalid; the rest are each transformed into a SAX word of *a* characters and length *p*.

Each section’s SAX word is compared using mindist to words created with the same *a* and *p* from a reference set of patterns. If at least 75% of the reference set words are equivalent to the section’s word, then the section is classified as a valid RSWC pattern. The whole process pseudo-code is shown in Algorithm 2.

**Algorithm 2** Series strings comparison.1:**function** (SSC(T,RefSet,a,p))
2:  valid_sections = list()
3:  valid_strings = list()
4:  **for**
*V* in RefSet **do**▹ Creates reference set’s strings5:   **Standardize**(V)
6:   string_V = **SAX_string**(V, a, p)
7:   valid_strings.append(string_V)
8:  **end for**
9:  **for**
section in *T*
**do**
10:   **if** basic_rules(section) **then**
11:    counter =0
12:    **Standardize**(section)
13:    string_section = **SAX_string**(section, alfa = a, paa_size = p)
14:    **for** string_V in valid_strings **do**
15:     **if equal_mindist**(string_V, string_section) **then**
16:      counter++
17:     **end if**
18:    **end for**
19:    **if** counter ≥0.75×length(RefSet)
**then**▹ 75% of the reference set matches20:     valid_sections.append(section)
21:    **end if**
22:   **end if**
23:  **end for**

   **return** (valid_sections)
24:**end function**


Both algorithms have linear complexity; each part of the algorithms is at most O(n), as demonstrated in their respective original papers [18,22,23]. SSC is considerably faster in execution and has the advantage that it can be used to identify a single section, whereas TRP needs the whole time series to function.

### 3.5. Ensemble Algorithms

It is hard to know beforehand which values the parameters should take when running the algorithms on data from a new field, especially since the data are not labeled and there is no way to verify if the identified patterns are correct.

In this case, there are three parameters, w,a and *p*. It was determined during initial testing that for the window size to discretize the series, a good option is to consider the average length of the sections of *T*. For the other two parameters, a simple ensemble learning algorithm is used to execute the algorithms using different combinations and to obtain a more accurate classification.

The result of one execution of the algorithm can be represented as a vector of length *s* where *s* is the number of sections in the dataset. Each element represents a section, and the corresponding algorithm assigns the values as −1 if the section is invalid or 1 if it is a valid pattern.

The ensemble algorithm runs SSC, TRP and preTRP using every possible combination of the parameters *a* and *p* in each range and sums the resulting vectors. The final classification depends on the sign of the elements of that vector; if an element is negative, then most iterations were classified as −1, and the section is considered invalid and the opposite for a positive element. For example, a range of 4 to 10 can be determined for the alphabet size *a* and from 4 to 12 for the word size *p*; this means the algorithm will be executed 63 times, and the final vector will have values within the [−63, 63] range.

### 3.6. Performance Metrics

In order to assess the performance, a confusion matrix is generated for each dataset and algorithm execution. Using the ground truth defined previously, it is possible to check whether a section was correctly classified or not by the algorithms. Table 4 shows a confusion matrix and its corresponding performance metrics. In this context, positive refers to a section being labeled as valid at the ground truth set; negative refers to a section being labeled as invalid at the ground truth set. The main metric to maximize is precision while achieving a low number of false positives.

(2) $$\begin{array}{cc} Accuracy=\frac{TP+TN}{P+N} & Precision=\frac{TP}{TP+FP}\\ Recall=\frac{TP}{P} & F1Score=\frac{2\times TP}{2\times TP+FP+FN} \end{array}$$

## 4. Results

In this section, the proposed algorithms’ (TRP, preTRP and SSC) results are exposed and compared to the current implemented DHC algorithm results. Result tables highlight the best algorithm for each metric when compared to the original DHC using a green or red arrow and showing the net difference for that metric. A confusion matrix for each algorithm is also displayed at the right side of each table.

The parameters were fine-tuned by testing all possible combinations of *a* and *p* between four and 18 for the TRP and preTRP algorithms and between four and nine for the SSC algorithm on the training sets (Avocados 1C, Avocados M3 and Kiwis 1A). The best results for TRP and preTRP came from combining both parameters in the range of four to 16; for SSC, it was determined that the best range was to use the combinations of *a* between six and eight and *p* in the range of four to nine. The parameter *w* for TRP and preTRP was picked as the average length of the sections in the time series; in the case of preTRP, the sections that are pre-classified with the basic rules are ignored for the determining of *w*.

The reference set for SSC corresponds to all of the manually-identified valid sections (ground truth) in the three training datasets. Using the algorithm with this reference set on the training sets could lead to altered results; however, different tests were carried excluding the patterns of the corresponding training set on the reference set, and the results were identical. This happens because having the correct pattern in the reference set for a section only secures one match, where 75% of the reference set is needed for a positive classification.

### 4.1. Training Datasets’ Results

#### 4.1.1. Avocados 1C

This field is the easiest to manually verify; most of the wrong patterns are visually obvious, and the rest can be discriminated using the DHC pre-classification rules explained in Section 3.3. The results for this dataset are shown in Table 5.

The DHC algorithm had good results on this field, achieving the highest recall (0.935) because sections where mainly within a constant soil moisture range; still, the only thing it does better than the others is identifying a greater number of patterns. The high recall achieved by the DHC algorithm comes with a cost of over 10 false positives and lower performance on all of the other metrics. The best results are from SSC; having a reference set gives this algorithm an advantage, and the other results are still comparable even when the classification is unsupervised.

#### 4.1.2. Avocados M3

Results for this dataset are shown in Table 6.

The performance of the DHC algorithm is the worst by far; this happened because of the upwards tendency on the humidity levels of the data. The DHC algorithm does not standardize sections when comparing, which greatly affects the matching of patterns when the reference set is not updated. In this case, the best results are once again achieved by SSC; the metric where it gets second place is recall. TRP identifies correctly the highest number of patterns (67 TP + 145 TN); the number of false positives (13 FP) is a bit higher, but it still maintains precision and F1 score over 0.80.

#### 4.1.3. Kiwis 1A

The Kiwis 1A field has a seasonal behavior, with most of the correct patterns happening during summer months: December, January, February and March. The DHC algorithm does a good job at finding the patterns, identifying most of the correct sections, but it labels almost as many incorrect patterns as valid. The results are shown at Table 7.

For this dataset, preTRP gets the best results; the number of false positives (four FP) is less than 10% of the false positives (47 FP) that the DHC algorithm achieved. Furthermore, preTRP achieves the highest metrics on almost all categories. TRP manages to identify every correct section while almost halving the number of false positives (24 FP) that the DHC algorithm had. SSC’s results are somewhere in between TRP and preTRP, but remain better than the DHC algorithm on every category.

### 4.2. Testing Datasets Results

#### 4.2.1. Nectarines W1

This dataset has only 27 valid sections and 117 invalid sections, obtained from the ground truth definition for this dataset, making it a highly imbalanced scenario. Results are shown in Table 8.

The DHC algorithm manages to identify all of the valid patterns; however, it also adds more than twice as many false positives (65 FP, 27 TP). All three newly-proposed algorithms drastically improve the number of false positives identified; a tendency starts to form on the algorithms’ results. TRP is usually the best at identifying valid sections, while SSC keeps the best precision score; preTRP stays in between the two for every metric.

**Figure 12:**
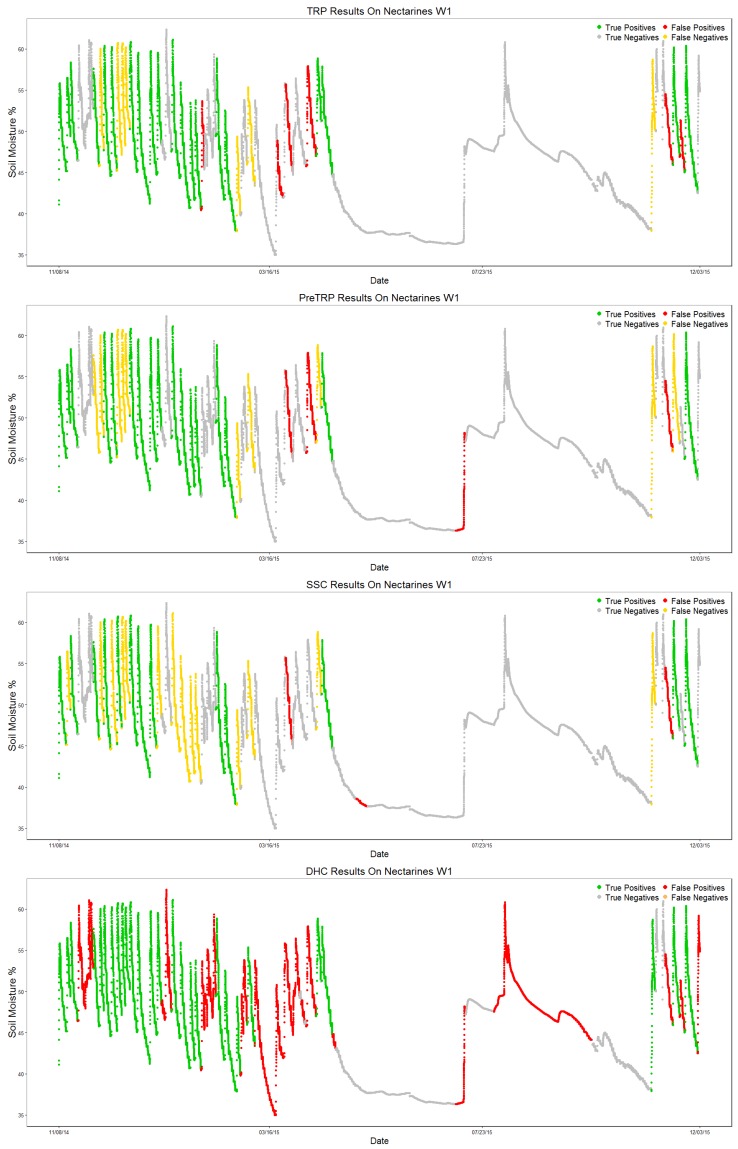
Plots of the output for each algorithm on the Nectarines W1 test dataset.

Figure 12 shows each algorithm’s output, labeling each section as True Positive (TP, green), True Negative (TN, gray), False Positive (FP, red) or False Negative (FN, yellow) by comparing the algorithm output to the ground truth, similar to the confusion matrix.

#### 4.2.2. Avocados A3

Results for this dataset are shown in Table 9.

The DHC algorithm once again identifies every valid pattern (1.0 recall); the number of false positives is still too high (61 FP). As expected, results show that the three newly-proposed algorithms have better results, especially when it comes to lowering the number of false positives: 10 FP for TRP, 3 FP for preTRP and 2 for SSC. TRP correctly identifies almost every valid pattern, generating less than 20% of the DHC algorithm’s false positive count. SSC maintains the highest precision score and has the lowest number of false positives (two FP); preTRP stays in between the other two algorithms in every metric, making it a good algorithm to balance the trade-off between precision and recall.

**Figure 13:**
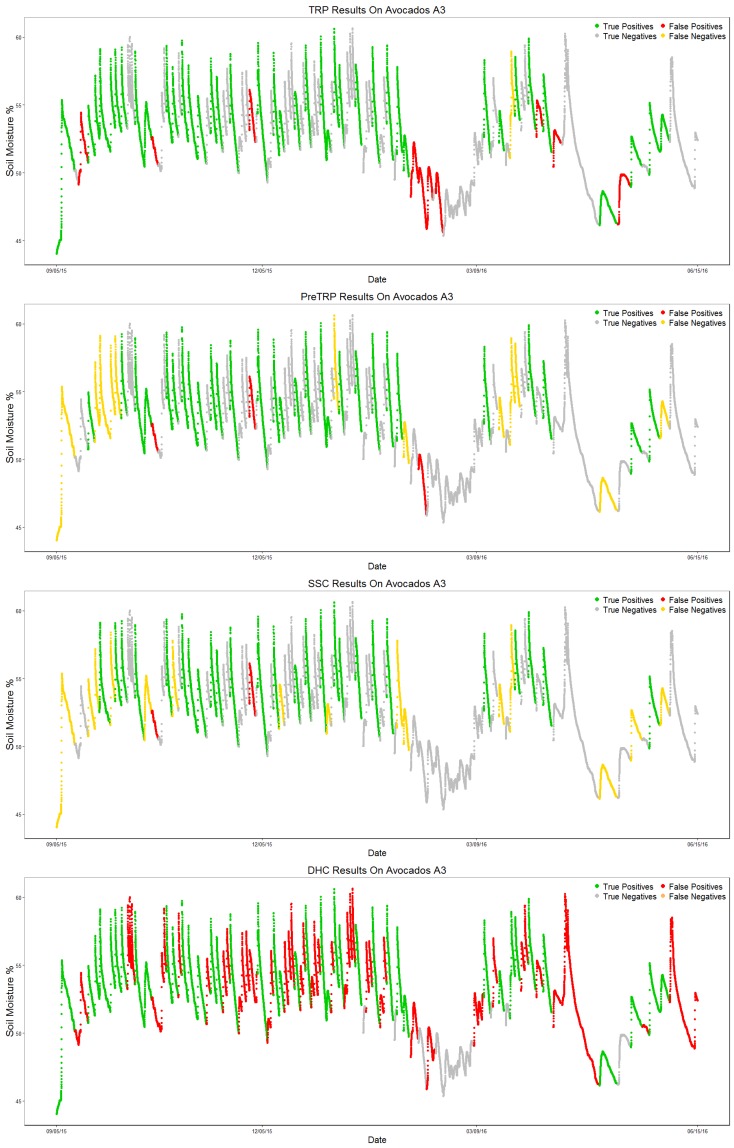
Plots of the output for each algorithm on the Avocados A3 test dataset.

Figure 13 shows each algorithm’s output, labeling each section as True Positive (TP, green), True Negative (TN, gray), False Positive (FP, red) or False Negative (FN, yellow) by comparing the algorithm output to the ground truth, similarly to the confusion matrix.

### 4.3. Execution Time Analysis

An execution time analysis was done for each of the datasets and algorithms. The study considers the time it takes to complete each iteration of the algorithm and the total time it takes to get the result. With the parameter combinations used, TRP and preTRP execute 121 iterations each, and SSC runs only 18.

The DHC algorithm was not measured for this comparison; however, it must be stated that the algorithm is faster than the others since it only compares to a sample of 50 sections and does not use an ensemble technique.

Tests were done on an HP ENVY dv6 laptop with an Intel Core i7-3630QM 2.40-GHz processor and 16 Gb RAM. The laptop runs on Windows 10; the code is written in R and was executed using RStudio Version 0.99.903. Results are shown in Table 10.

Results show that SSC is by far the fastest algorithm, with an execution time under 5% the time for the TRP or preTRP algorithms. On the biggest dataset, it takes under a minute to get the final results (0:52). This result is as expected; the algorithm is simpler, and it requires less iterations. TRP and preTRP have similar execution times. In theory, preTRP should be faster especially on fields where most sections are pre-classified; however, empirically, the results vary when compared to TRP. The reason for this variation may be due to the conditions of the tests; other processes were running when the performance was evaluated, and this affected the results. While the results may not be exact, they give enough information to show how much faster SSC is in comparison to TRP and preTRP. Results also help to prove that there is no exponential increase in the time it takes to run the algorithms, empirically testing their linear complexity.

## 5. Discussion

In this work, we have presented three novel algorithms for identifying the RSWC from soil moisture sensor time series data. This pattern is not regular; it has different durations and can span different soil moisture ranges. The algorithm parameters were calibrated using three datasets, and testing was done using two other datasets. For assessing each method’s performance, an expert manually labeled the time series identifying which sections showed RSWC and which did not, then each algorithm classification output was compared to these ground truths.

The RSWC pattern is defined as the pattern generated by sensor measurements during irrigation and water consumption by crops. This pattern is composed by three consecutive segments: irrigation, fall and consumption. This pattern may be generated by on demand irrigation or due to rain fall. This study does not cover an analysis of these two causes; both are considered to have a similar effect. The correct identification of RSWC aids agronomists to quickly visualize expected irrigation behavior and identify pattern changes. The RSWC pattern is fundamental to learn the behavior of soil moisture mechanics and crop water consumption.

Three novel algorithms were developed to solve the problem: Top Rule Pattern (TRP), pre-Validated Top Rule Pattern (preTRP) and Series String Comparison (SSC); all of these algorithms are based on the Symbolic Aggregate ApproXimation (SAX) representation approach to time series analysis. The algorithms were compared to an actual deployed algorithm, Density Histogram Comparison (DHC). The three proposed algorithms can identify the RSWC patterns in the data and drastically improve the results obtained by the DHC algorithm by achieving a decrease in false positives rate (SSC mean FPR 0.026, DHC mean FPR 0.684) and an increase in precision (SSC average precision 0.872, DHC average precision 0.347).

TRP and preTRP are both unsupervised learning algorithms; their main disadvantage is the speed (over 9 min on average each); in order to get the best results, they require many iterations. Another issue is that they need to evaluate the whole time series before obtaining an output; they cannot identify if a single section is a valid pattern because they must generate rules based on the whole time series data. TRP achieved a mean precision of 0.785 and mean FPR of 0.092; preTRP achieved a mean precision of 0.858 and mean FPR of 0.037. SSC is the fastest of the three newly-proposed algorithms, under 20 s on average. It also allows for online identification of sections since it does not need to evaluate the whole time series data. SSC achieved the highest mean precision of 0.872 and the lowest mean FPR of 0.026.

SSC is the best performing algorithm. The main objective was to minimize false positives, and SSC is the best at that; it also has the advantage of being able to process new sections as they are added to the data without using the need to revise the previous time series data. Despite being a very simple algorithm, SSC reaffirms the capabilities of SAX representation [22] for comparing time series data, and it was demonstrated that it can be applicable to new datasets where there are no training data available using reference patterns from a different field.

Further work may address with detail the reasons why some time series sections were labeled as invalid (not RSWC), adding context information and sensors such as weather stations, the GPS position of workers and machinery, crop phenology stage, among others. All of these sensors may add more information regarding why the soil moisture behaves that way. From the point of view of computer science, it is interesting to research an artificial intelligence algorithm that can automatically determine the best parameter setting for each algorithm. This automatic determination of parameters would allow the algorithm to be easily adaptable to similar time series data problems.

## Figures and Tables

**Table 1:** Training datasets’ summary.

Field	Measurements	Duration (Days)	Sections	Valid	Invalid	Pre-Classified
Avocados 1C	28,026	291.9	88	62	26	18
Avocados M3	43,649	454.6	230	72	158	141
Kiwis 1A	66,638	694.1	425	56	369	329

**Table 2:** Testing datasets’ summary.

Field	Measurements	Duration (Days)	Sections	Valid	Invalid	Pre-Classified
Nectarines W1	36,802	383.3	144	27	117	74
Avocados A3	26,183	272.7	116	41	75	54

**Table 3:** Breakpoints that divide a N(0,1) distribution on equiprobable regions.

$\beta_{i}\backslash \alpha$	3	4	5
$\beta_{1}$	−0.43	−0.67	−0.84
$\beta_{2}$	0.43	0	−0.25
$\beta_{3}$	-	0.67	0.25
$\beta_{4}$	-	-	0.84

**Table 4:** Confusion matrix and performance metrics.

		Prediction		
		Positive	Negative	Total
**Truth**	**Positive**	True Positive (TP)	False Negative (FN)	Positive
	**Negative**	False Positive (FP)	True Negative (TN)	Negative
	**Total**	TP+FP	FN+TN	

**Table 5:** Final results for the field Avocados 1C.

Avocados 1C								
Algorithm	Accuracy	Precision	Recall	F1 Score			Prediction	
							1	0
TRP	0.864	0.946	0.855	0.898	Truth	1	53	9
						0	3	23
preTRP	0.852	0.962	0.823	0.887	Truth	1	51	11
						0	2	24
SSC	$\overset{▴0.10}{0.886}$	$\overset{▴0.17}{0.964}$	$\overset{▾0.06}{0.871}$	$\overset{▴0.06}{0.915}$	Truth	1	54	8
						0	2	24
DHC	0.784	0.795	0.935	0.859	Truth	1	58	4
						0	15	11

**Table 6:** Final results for the field Avocados M3.

Avocados M3								
Algorithm	Accuracy	Precision	Recall	F1 Score			Prediction	
							1	0
TRP	0.922	0.838	$\overset{▴0.65}{0.931}$	0.882	Truth	1	67	5
						0	13	145
preTRP	0.896	0.875	0.778	0.824	Truth	1	56	16
						0	8	150
SSC	$\overset{▴0.43}{0.930}$	$\overset{▴0.66}{0.900}$	0.875	$\overset{▴0.63}{0.887}$	Truth	1	63	9
						0	7	151
DHC	0.496	0.238	0.278	0.256	Truth	1	20	52
						0	64	94

**Table 7:** Final results for the field Kiwis 1A.

Kiwis 1A								
Algorithm	Accuracy	Precision	Recall	F1 Score			Prediction	
							1	0
TRP	0.944	0.700	$\overset{▴0.09}{1.000}$	0.824	Truth	1	56	0
						0	24	345
preTRP	$\overset{▴0.09}{0.965}$	$\overset{▴0.40}{0.918}$	0.804	$\overset{▴0.20}{0.857}$	Truth	1	45	11
						0	4	365
SSC	0.948	0.827	0.768	0.796	Truth	1	43	13
						0	9	360
DHC	0.878	0.520	0.911	0.662	Truth	1	51	5
						0	47	322

**Table 8:** Final results for the field Nectarines W1.

Nectarines W1								
Algorithm	Accuracy	Precision	Recall	F1 Score			Prediction	
							1	0
TRP	$\overset{▴0.36}{0.910}$	0.769	$\overset{▾0.26}{0.741}$	$\overset{▴0.30}{0.755}$	Truth	1	20	7
						0	6	111
preTRP	0.903	0.810	0.630	0.708	Truth	1	17	10
						0	4	113
SSC	0.882	$\overset{▴0.52}{0.813}$	0.481	0.605	Truth	1	13	14
						0	3	114
DHC	0.549	0.293	1.000	0.453	Truth	1	27	0
						0	65	52

**Table 9:** Final results for the field Avocados A3.

Avocados A3								
Algorithm	Accuracy	Precision	Recall	F1 Score			Prediction	
							1	0
TRP	$\overset{▴0.43}{0.905}$	0.800	$\overset{▾0.02}{0.976}$	$\overset{▴0.31}{0.879}$	Truth	1	40	1
						0	10	65
preTRP	0.871	0.906	0.707	0.795	Truth	1	29	12
						0	3	72
SSC	0.862	$\overset{▴0.53}{0.931}$	0.659	0.771	Truth	1	27	14
						0	2	73
DHC	0.474	0.402	1.000	0.573	Truth	1	41	0
						0	61	14

**Table 10:** Performance results. Each iteration is the time it takes to get the results for one set of parameters.

		Avocados 1C	Avocados M3	Kiwis 1A	Nectarines W1	Avocados A3
	Measurements	28.026	43.649	66.638	36.802	26.183
	Sections	88	230	425	144	116
	Pre-Classified	18	141	329	74	54
**TRP**	Iteration time (s)	3.176	4.780	9.859	3.510	2.089
	Total time (min)	6:32	9:40	19:56	7:06	4:14
**preTRP**	Iteration time (s)	4.066	5.003	8.967	2.606	2.251
	Total time (min)	8:13	10:07	18:06	5:16	4:34
**SSC**	Iteration time (s)	0.484	1.005	2.865	0.493	0.470
	Total time (min)	0:08	0:18	0:52	0:09	0:09

## Abbreviations

The following abbreviations are used in this manuscript:

RSWC	Root System Water Consumption
FDR	Frequency Domain Reflectometry
DHC	Density Histogram Comparison
SAX	Symbolic Aggregate Approximation
PAA	Piecewise Aggregation Approximation
TRP	Top Rule Patterns
preTRP	pre-classified Top Rule Patterns
SSC	Series Strings Comparison
